# Targeted Radiation Exposure Induces Accelerated Aortic Valve Remodeling in ApoE^−/−^ Mice

**DOI:** 10.3390/jcm12185854

**Published:** 2023-09-08

**Authors:** Guillaume Rucher, Kevin Prigent, Christophe Simard, Anne-Marie Frelin, Maëlle Coquemont-Guyot, Nicolas Elie, Nicolas Delcroix, Nicolas Perzo, Romain Guinamard, Ludovic Berger, Alain Manrique, on behalf of the STOP-AS Investigators

**Affiliations:** 1 Normandie Univ, UNICAEN, UR 4650 PSIR, GIP Cyceron, 14000 Caen, Francekevin.prigent.lp@gmail.com (K.P.); christophe.simard@unicaen.fr (C.S.); romain.guinamard@unicaen.fr (R.G.); berger-l@chu-caen.fr (L.B.); 2 Department of Nuclear Medicine, CHU de Caen, 14000 Caen, France; 3 Grand Accélérateur National d’Ions Lourds (GANIL), CEA/DRF-CNRS/IN2P3, 14000 Caen, France; 4 Normandie Univ, UNICAEN, SF 4207, PLATON Services Unit, Virtual’His, 14000 Caen, Francenicolas.elie@unicaen.fr (N.E.); 5 CNRS, UMS-3048, GIP Cyceron, Campus Jules Horowitz, 14000 Caen, France; 6 Normandie Univ, UNIROUEN, INSERM U1096 EnVI, 76000 Rouen, France; nperzo@gmail.com; 7 Department of Vascular Surgery, Normandie Univ, UNICAEN, UR 4650 PSIR, CHU de Caen, 14000 Caen, France

**Keywords:** aortic stenosis, radiation therapy, mineralization, magnetic resonance imaging

## Abstract

Thoracic radiation therapy may result in accelerated atherosclerosis and in late aortic valve stenosis (AS). In this study, we assessed the feasibility of inducing radiation-induced AS using a targeted aortic valve irradiation (10 or 20 Grays) in two groups of C57Bl6/J (WT) and ApoE^−/−^ mice compared to a control (no irradiation). Peak aortic jet velocity was evaluated by echocardiography to characterize AS. T2*-weighted magnetic resonance imaging after injection of MPIO-αVCAM-1 was used to examine aortic inflammation resulting from irradiation. A T2* signal void on valve leaflets and aortic sinus was considered positive. Valve remodeling and mineralization were assessed using von Kossa staining. Finally, the impact of radiation on cell viability and cycle from aortic human valvular interstitial cells (hVICs) was also assessed. The targeted aortic valve irradiation in ApoE^−/−^ mice resulted in an AS characterized by an increase in peak aortic jet velocity associated with valve leaflet and aortic sinus remodeling, including mineralization process, at the 3-month follow-up. There was a linear correlation between histological findings and peak aortic jet velocity (r = 0.57, *p* < 0.01). In addition, irradiation was associated with aortic root inflammation, evidenced by molecular MR imaging (*p* < 0.01). No significant effect of radiation exposure was detected on WT animals. Radiation exposure did not affect hVICs viability and cell cycle. We conclude that targeted radiation exposure of the aortic valve in mice results in ApoE^−/−^, but not in WT, mice in an aortic valve remodeling mimicking the human lesions. This preclinical model could be a useful tool for future assessment of therapeutic interventions.

## 1. Introduction

Cardiac diseases are a major cause of late mortality and morbidity after mediastinal radiotherapy. The delayed cardiotoxicity may involve all components of the heart, increasing the risk of pericardial disease, cardiomyopathy, arrhythmias, coronary artery disease, and valvular heart disease, including both aortic regurgitation and stenosis [1]. Radiation-induced aortic valve remodeling is characterized by fibrosis and calcification leading to aortic valve stenosis (AS) and/or regurgitation [1]. Although aortic regurgitation is one of the most common side effects, aortic stenosis is more likely to require an interventional correction [1]. Degenerative AS is a common form of valvular heart disease in developed countries [2], affecting 40% of patients over 80 years old [3]. After mediastinal radiotherapy, the progression of a pre-existing AS is accelerated, further underlining the potential of irradiation to initiate and develop valvular lesions [4]. The same phenomenon is also observed at the vascular level, with an acceleration and/or induction of atherosclerosis in irradiated patients [5].

Previous results have indicated that valve remodeling is mostly due to a direct effect of radiation on valvular cells. The incidence of valvular disease correlates with radiation dose directly delivered to the valve [6]. After an early inflammatory phase occurring within days of irradiation, fibrogenic effector cells can differentiate into myofibroblasts, characterized by collagen and α-smooth muscle actin secretion, which may lead to late fibrosis [7]. We recently observed that transient receptor potential melastatin 4 (TRPM4), a non-selective cation channel involved in the differentiation of human atrial fibroblasts into myofibroblasts [8], is also involved in irradiation-induced aortic valve fibrosis [9]. In addition, recent in vitro experiments demonstrated that irradiation of human aortic valvular interstitial cells (hVICs) induced the expression of osteogenic factors 24 h after irradiation, including bone morphogenetic protein 2 (BMP-2), alkaline phosphatase (ALP), and Runx2 [10]. These phenomena are similar to the early pathophysiology of degenerative AS which involves mechanical lesions, chronic inflammation, and osteogenic phenotypic changes in valve interstitial cells, leading to progressive mineralization [11].

The combination of cardiovascular risk factors and radiation therapy increases radiation-induced peripheral atherosclerosis in humans [12]. The apolipoprotein E-deficient (ApoE^−/−^) mouse is a widely used animal model of atherosclerosis, demonstrating spontaneous atherosclerotic lesions throughout the aortic tree [13] and late aortic valve sclerosis similar to that observed in humans [14]. Based on these findings, we hypothesized that a targeted aortic valve irradiation in ApoE^−/−^ mice may induce an accelerated valve remodeling mimicking human delayed radiation-induced aortic valve stenosis.

## 2. Materials and Methods

### 2.1. Animals

The control animals were distributed in 2 groups of C57Bl6/J (group WT RT-, n = 10) and ApoE^−/−^ mice (group ApoE^−/−^ RT-, n = 11). A total of 26 animals were randomly allocated to 10 or 20 Grays (Gy) for both genotypes (WT 10 Gy, n = 6; WT 20 Gy, n = 6; ApoE^−/−^ 10 Gy, n = 4; and ApoE^−/−^ 20 Gy, n = 10). All animals were male and maintained ad libitum with a standard chow diet composed of 8.4% fat, 19.3% protein, 72.4% carbohydrates, 0.55% phosphorus, 0.73% calcium, 0.16% magnesium, and 1000 UI/kg vitamin D3.

### 2.2. Three-Dimensional Anatomic Atlas of Aortic Valve

To target aortic valve irradiation, we built a murine cardiac atlas using cardiac magnetic resonance (CMR). All CMR experiments were carried out using a 7T magnet (Pharmascan^®^ Bruker, Billerica, MA, USA) under gas anesthesia induced with 5% isoflurane (Forene^®^, AbbVie, Rungis, France) and maintained with 2% isoflurane in a mix of O_2_ and N_2_O (1:2). T1 Flash MR images were performed in 13 male C57Bl6/J mice (16 weeks old) using a strict axial multi-slice sequence encompassing the heart: TR/TE 176.6/4.2 ms, 20 slices, 6 repetitions. Automatic alignment and fusion of the 6 repetitions were performed using ImageJ software (version 1.52a, Bethesda, MD, USA) to obtain one single image series for each animal. The aortic valve and the aorta from the insertion to the aortic isthmus were segmented. Then, a threshold set to 75% of co-localization of each aortic valve segmentation was performed to determine the aortic valve segmentation on the atlas.

### 2.3. In Vivo Irradiation Protocol

**Figure 1:**
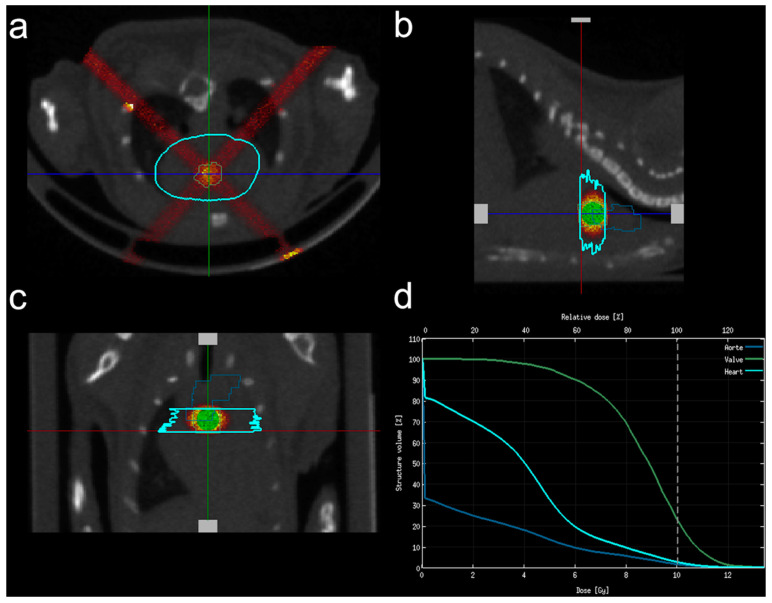
Irradiation plan consisted of 2 X-ray beams of 2 mm diameter with an angle of 45° and 135°. (**a**) Axial view; (**b**) sagittal view; (**c**) frontal view; (**d**) relative dose calculation within the aortic valve (green line), the surrounding heart (light blue) and the aortic arch (dark blue).

All aortic valve irradiations were performed in 16-week-old animals at the EquipHex RecHadron facility (Caen, France), with an X-RAD 225Cx micro-irradiator (Precision X-ray Inc., North Brandord, CT, USA) using fractions of 2.22 Gy/min (225 kV, 13 mA, 0.3 mm copper filter). Anesthesia was induced with 5% isoflurane (Forene^®^, AbbVie, Rungis, France) and maintained with 2% isoflurane in a mix of O_2_ and N_2_O (1:2). Animals were placed in prone position on a dedicated bed and computed tomography (CT) encompassing the chest (80 kV, 0.5 mA) was performed. Using 3D Slicer v4.8.1 software (http://www.slicer.org, accessed on 1 June 2018), the segmented aortic valve provided by the cardiac atlas was aligned with CT acquisition using trachea and bronchia bifurcation as anatomical markers. Planning of radiation exposure was performed using SmartPlan^®^ (Precision X-ray Inc., North Brandford, CT, USA). Tissues (air, lung, soft tissue, bone) were segmented using Hounsfield units. Irradiation consisted of 2 beams of 2 mm diameter with an angle of 45° and 315° (Figure 1) in order to avoid both the trachea and the esophagus, after beam spatial resolution and dose distribution were confirmed using polymethyl methacrylate (PMMA) phantoms and gafchromic films.

### 2.4. Echocardiography

Echocardiography was performed in isoflurane-anesthetized mice using a iE33 ultrasound system (Philips Healthcare, Best, The Netherlands) and a linear ultrasound probe L15-7io (128 elements, 7–15 MHz). M-mode images of the parasternal long and short axis views were used at baseline and 3-month follow-up to measure left ventricle dimensions. Aortic valve function was assessed using a Doppler measurement of peak aortic jet velocity and mean transvalvular gradient. The mean of 3 consecutive measurements for each parameter was calculated.

### 2.5. Magnetic Resonance Imaging of Aortic Inflammation

Additional MR experiments were carried out using a 7T magnet (Pharmascan^®^ Bruker, Ettlingen, Germany). End-diastolic MR images were acquired using a multi-slice T2*-weighted sequence encompassing the thoracic aorta in 2 WT RT-, 4 WT 10 Gy, in 3 WT 20 Gy, in 4 ApoE^−/−^ RT-, in 4 ApoE^−/−^ 10 Gy, and in 6 ApoE^−/−^ 20 Gy mice. The acquisition parameters were as follows: field of view: 1809 × 939 mm, slice thickness: 0.15 mm, spatial resolution: 0.1 × 0.1 mm, TR/TE: 100/4.25 ms. Acquisitions were performed before and after intravenous injection of 200 µL of MPIO-αVCAM-1. As previously described [15], microparticles of iron-oxide (DynaBeads MyOnes Tosyl Activated, ThermoFisher Scientific, Waltham, MA, USA) were conjugated with the antibodies anti-αVCAM-1 (clone A(429), BD BioScience, Franklin Lakes, NJ, USA) through incubation at 37 °C for 48 h. MR images were analyzed using Osirix v.6.5.2 software. A T2* signal void on valve leaflets and aortic sinus resulting from MPIO-αVCAM-1 binding was considered positive.

### 2.6. Histological Analysis

After completion of the study, mice were killed and perfused with heparinized (50 U/mL) phosphate-buffered saline 5/100 (PBS). Then, the heart was harvested and cryomounted in optimal cutting embedding medium (CellPath, ThermoFisher Scientific, Waltham, MA, USA). For each animal, eight 10 µm thick slices were collected. Cryosections were placed in 5% silver nitrate solution for 30–60 min then fixed in 5% sodium-thiosulfate solution for 2–3 min. Sections were parallel to the valve plane and digitized using ScanScope CS (Leica Biosystems, Wetzlar, Germany). Regions of interest (ROI) encompassing the valve leaflets and the aortic sinus were manually drawn using Aperio ImageScope software v12.3 (Leica Biosystem, Wetzlar, Germany). The valvular ROIs were processed to detect the rate of von Kossa staining, using Python programing language (Python Software Foundation, www.python.org, accessed on 29 November 2018) and OpenSlide [Geospatial Data Abstraction Library (GDAL) and Mahotas] for image processing [16]. The aortic valve leaflet area, the area of aortic sinus tissue, and the percentage of von Kossa staining within the aortic valve leaflets were assessed. Results were expressed as the relative proportion of stained tissue to total tissue area.

### 2.7. Irradiation of Human Aortic Valvular Interstitial Cells

As the valvular interstitial cells (hVICs) play a central role in the aortic valve mineralization, we also investigated the possible negative impact of irradiation on the viability of hVICs. Human aortic tricuspid valves were collected anonymously from patients with calcific aortic valve disease undergoing valve replacement surgery at Rouen University Hospital (Rouen, France). In accordance with French legislation, the patients gave their informed consent to participation. The study was approved by the regional ethics committee (Comité de Protection des Personnes Nord Ouest I, Rouen, France, 2 May 2016) and the patients provided informed consent. The hVICs were isolated from non-calcified areas of the valves as previously described [17,18]. Gibco™ Dulbecco’s modified Eagle’s medium (DMEM), high glucose with Gluta-MAX™ 11574456 (Fisher Scientific™) supplemented with 10% fetal bovine serum (FBS) 11573397 (Fisher Scientific™), and 1% antibiotics (100 IU/mL penicillin-G-Na; 50 IU/mL streptomycin sulfate) was used for cell culture. Experiments were performed on cells, in T25 flasks, from passages 2 to 4. Irradiations (10 or 20 Gy) were performed using the same Pxi225CX micro-irradiator.

The cell viability of hVICs was studied by flow cytometry. Twenty-four hours after irradiation, the supernatant was collected and cells were resuspended by trypsination. Supernatant and cells were centrifuged and incubated for 10 min in PBS solution with 20 µg/mL of propidium iodide. Propidium iodide staining was analyzed by the Cytoflex-GalliosTM flow cytometer (Beckman Coulter SAS, Marseille, France). The number of viable cells in each culture after irradiation was achieved based on the CytExpert 2.4 Flow Analysis software (Beckman Coulter SAS, Marseille, France).

Twenty-four hours after irradiation protocol, the cells were washed with cold PBS, and resuspended by trypsination. Cells were fixed in 70% ethanol solution. The cell cycle of hVICs was studied by flow cytometry with a classical propidium iodide (50 µg/mL, life technologies, Carlsbad, CA, USA) solution with RNase A (20 mg/mL, life technologies) in PBS. Propidium iodide staining was analyzed by the Cytoflex-GalliosTM flow cytometer (Beckman Coulter SAS, Marseille, France). The analysis and determination of the cell distribution in each phase of the cell cycle was achieved based on the Kaluza^®^ Flow Analysis software (Beckman Coulter SAS, Marseille, France).

### 2.8. Statistical Analysis

Values were expressed as mean ± SEM. A linear model analysis was used to evaluate the effect of the time, the genotype, and the radiation dose. A post hoc analysis was performed using Tukey HSD test only when the lineal model was significant. Otherwise, i.e., when the global *p*-value for the linear model was not significant, no post hoc effect was performed. A linear regression was used to correlate the quantitative analysis of von Kossa staining and sinus lesion area with peak aortic jet velocity. For proportions, the Fischer exact test was used to compare differences between groups. Statistical analyses were performed using JMP 11 (SAS Institute, Cary, NC, USA), and a *p* value ≤ 0.05 was considered statistically significant.

## 3. Results

Radiation exposure was well tolerated in all animals.

### 3.1. Echocardiography

In ApoE^−/−^ mice, there was an impairment of left ventricular function compared to WT mice, as demonstrated by increased LVDs resulting in a decreased LV fractional shortening (see Table 1). There was no effect of radiation exposure on left ventricular function over time. On the other hand, the peak aortic jet velocity was significantly higher at the 3-month follow-up in ApoE^−/−^ mice (*p* < 0.0001) compared to WT.

**Figure 2:**
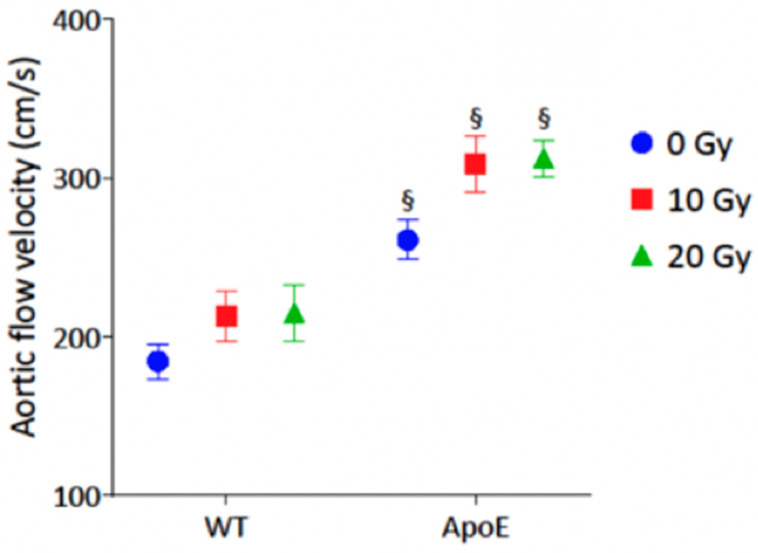
Impact of radiation exposure dose on peak aortic jet velocity at 3-month follow-up. The analysis was performed in WT control (n = 10), WT 10 Gy (n = 6), WT 20 Gy (n = 6), ApoE^−/−^ control (n = 11), ApoE^−/−^ 10 Gy (n = 4), and ApoE^−/−^ 20 Gy (n = 10). Data are expressed in mean ± SEM, § *p* < 0.05 vs. dose-equivalent WT.

As shown in Figure 2 and Table 2, radiation exposure resulted in a further increase in peak aortic jet velocity at 3 months (*p* < 0.001), suggesting a radiation-induced aortic valve remodeling.

### 3.2. α-VCAM MPIO MR Imaging Findings

Twenty-three animals (WT: n = 9, ApoE^−/−^: n = 14) underwent α-VCAM MPIO MR imaging. A T2* signal void was noted in the aortic sinus in 15/23 (65%) cases and in the aortic valve leaflets in 18/23 (78%) cases (Table 3).

**Figure 3:**
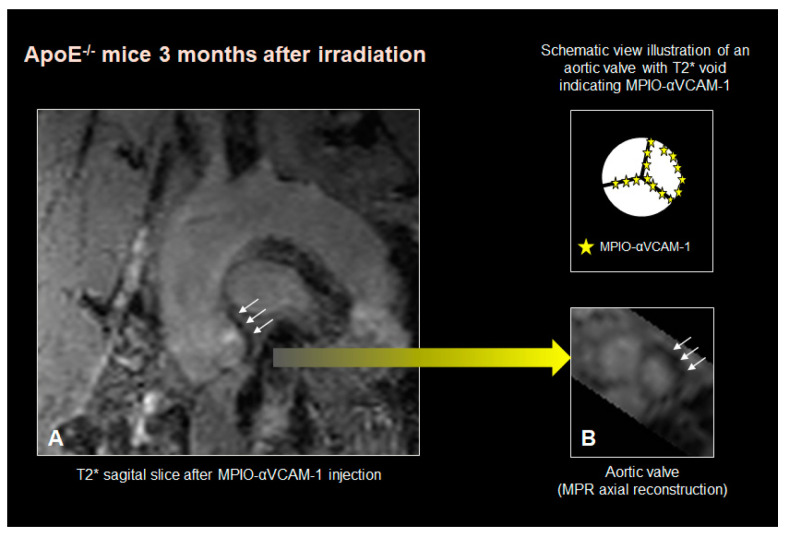
Example of a T2* signal void involving both the aortic sinus and the valve leaflets in an ApoE^−/−^ mice imaged 3 months after irradiation. (white arrows indicating the signal void). (**A**): sagittal view, (**B**): short axis reconstruction of the aortic valve with the corresponding scheme depicting the MPIO-αVCAM–1 (yellow stars) on the valve leaflets.

Figure 3 depicts a T2* signal void involving both the aortic sinus and valve leaflets in an ApoE^−/−^ mice imaged 3 months after irradiation. The association of the T2* signal void with radiation exposure reached statistical significance within the aortic valve leaflets (Fischer exact test *p* < 0.01), but not within the aortic sinus, suggesting a specific impact of targeted irradiation on the expression of VCAM-1 within the aortic valve endothelium.

### 3.3. Histological Analysis

Histological findings showed that, independently of radiation exposure, ApoE^−/−^ mice showed an aortic sinus thickening demonstrated by an increased aortic sinus tissue area compared to WT (Table 4). 

**Figure 4:**
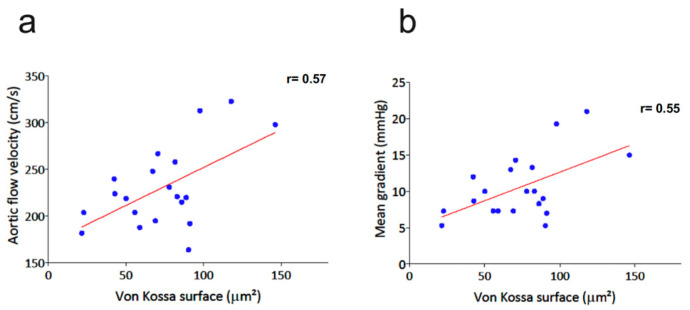
Correlation of von Kossa staining with peak aortic jet velocity (**a**) and mean trans-valvular gradient (**b**) in the whole study population.

In addition, there was a significant remodeling of valve leaflets and aortic sinus wall related to radiation exposure, as demonstrated by the increased tissue area, especially in ApoE^−/−^ mice. This remodeling further increased with the radiation dose in ApoE^−/−^. Von Kossa staining showed that radiation exposure promoted a mineralization process in the valve leaflets (*p* < 0.001), especially in ApoE^−/−^ mice. It is worth noting that the mineralization process was decreased in ApoE^−/−^ after 20 Gy compared to 10 Gy. As described in Figure 4, there was a significant correlation of von Kossa staining with peak aortic jet velocity (r = 0.57, *p* < 0.01) and mean trans-valvular gradient (r = 0.55, *p* = 0.02).

### 3.4. hVICs Analysis

**Figure 5:**
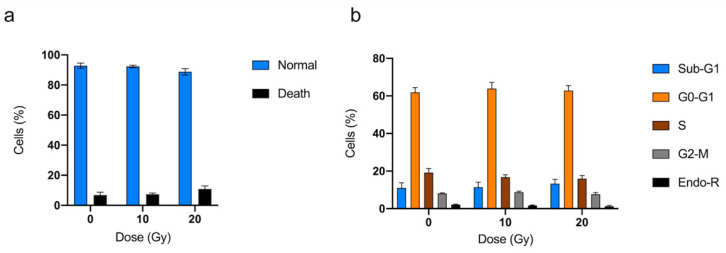
Impact of radiation exposure on isolated valve interstitial cells from patients using viability (**a**) and cell cycle analysis (**b**). Sub-G1: Sub-growth-phase-1; G0-G1: growth 0 and growth 1 phase; S: synthesis phase; G2-M: growth 2 phase and mitosis; Endo-R: Endo-replication. Data are expressed in mean ± SEM.

Cell cycle and viability analyses of hVICs were performed in, respectively, four and five experiments under control conditions, and 10 Gy and 20 Gy under irradiation conditions. Radiation exposure did not reduce the viability of hVICs, and had no impact on cell cycle analysis (Figure 5).

## 4. Discussion

The main result of this study is that in ApoE^−/−^ mice, a targeted aortic valve irradiation resulted in an aortic valve remodeling demonstrated by a significant increase in peak aortic jet velocity at a 3-month follow-up, an effect that was not significant in wild type mice.

Degenerative calcified AS is characterized by fibro-calcific remodeling of the valve leaflets. The progression of the disease involves severe calcification within the valve leaflets, leading to an impairment of valve motion contributing to blood flow obstruction [11]. A high incidence of valvular dysfunction has been reported in populations undergoing mediastinal radiation therapy [19]. In these patients, the progression of a pre-existing AS is accelerated, underlining the potential of X-rays to initiate and develop valvular lesions [4]. Previous results in patients requiring valve surgery described specific features of radiation-induced valve lesions, including various levels of diffuse leaflet fibrosis and retractions, which differentiate these radiation-induced lesions from degenerative calcified valvular stenosis [20].

In vitro studies emphasized the relationship between irradiation and the behavior of hVICs, which are the main cell type in aortic valve cusps. Early results showed that a 10 Gy irradiation of hVICs induced an osteogenic phenotype differentiation demonstrated by a significant increase in bone morphogenetic protein 2, osteopontin, alkaline phosphatase, and Runx2 [10]. In addition, low-dose-radiation exposure of porcine valvular interstitial cells resulted in myfibroblast-like changes associated with calcification, while high doses equivalent to 60 Gy over 30 fractions produced DNA damage leading to a decrease in cell viability [21]. In the present study, we found no reduction in cell viability and no impact on cell cycle in hVICs exposed to 10 Gy or to 20 Gy. Although valve remodeling increased with the radiation dose, as demonstrated by the increased amount of tissue within the aortic sinus, increasing radiation from 10 to 20 Gy in ApoE^−/−^ mice did not result in a further increase in aortic peak velocity. This is in agreement with previous findings by Meerman et al. [21], who found that calcification, assessed by alkaline phosphatase activity, was mostly present in VICs exposed to 4 Gy, while higher doses equivalent to 60 Gy resulted in giant fibroblast-like cell changes. Our findings are in agreement with these results, suggesting that low-dose irradiation (up to 20 Gy) may induce an osteogenic transition without cell death. This is also in agreement with clinical findings demonstrating a mix of fibrosis and calcifications in patients with a history of mediastinal radiation therapy [20].

In a recent study, Mpweme et al. [9] demonstrated that radiation-induced aortic valve remodeling was inhibited in TRPM4^−/−^ mice. TRPM4 is a monovalent non-selective cation channel involved in calcium handling and participating in fibroblast transition to myofibroblasts, a phenomenon observed during aortic valve stenosis. In their study, maximal aortic valve jet velocity was evaluated at a 5-month follow-up and was significantly higher in irradiated compared to non-irradiated wild type Trpm4^+/+^ mice (240.9 ± 17.2 and 185.1 ± 7.9 cm·s^−1^, respectively), while no difference was observed in Trpm4^−/−^ animals depending on irradiation. The authors also noted that irradiation induced a significant increase in cusp surface in Trpm4^+/+^ mice compared to Trpm4^−/−^ animals, as well as in the total surface of the cusp and aortic annulus, with a linear correlation between these pathological findings and maximal aortic jet velocity. Compared to this latter study, we found a slightly lower aortic jet velocity at the 3-month follow-up after radiation exposure in WT animals, whereas it was further increased in ApoE^−/−^. This aortic valve remodeling in ApoE^−/−^ mice was associated with valve mineralization that was not observed in WT mice, in agreement with previous findings from Mpweme et al. [9]. In addition, there was a significant relationship between von Kossa staining and peak aortic jet velocity. These results demonstrated that, compared to WT animals, ApoE^−/−^ mice are more likely to develop accelerated aortic lesions after irradiation, as demonstrated by ultrasound and histological findings.

In humans, the risk of radiation-induced peripheral atherosclerosis is significantly increased when cardiovascular risk factors are combined with radiation therapy [12]. Although there is a lack of research documenting the impact of combined risk factors and radiation on the occurrence of aortic stenosis, it has been demonstrated that pre-existing risk factors are strongly associated with severe calcific AS [22]. Similarly, calcific deposition has been previously reported in a mouse model of high-fat-diet-induced AS [23,24]. In addition, valve leaflet thickening was also found in Ldlr^−/−^ Apob100/100 mice with a 0.15% cholesterol diet, associated with an increase in peak aortic jet velocity which was rescued by a regular exercise training [25]. To our knowledge, our study is the first preclinical investigation evaluating the impact of targeted radiation exposure on the development of AS in ApoE^−/−^ mice. Previous results showed that C57BL/6J mice fed with a Western diet may demonstrate inflammatory features similar to early atherosclerotic lesions [26]. ApoE^−/−^ mice of C57BL/6 background develop atherosclerosis throughout the arterial tree, including the aortic root at the base of the valve, and these lesions, a condition that favors vascular inflammation, are accelerated when mice are fed with a Western diet [13]. Using echocardiography and histologic examination, Tanaka et al. [14] documented sclerotic changes associated with functional abnormalities in senile ApoE^−/−^ mice yielding aortic valve sclerosis that was similar to the results we observed in younger individuals after targeted aortic valve irradiation. These results are in line with human investigations demonstrating an acceleration of aortic valve lesions after thoracic irradiation [20].

As irradiation in itself induces local inflammation, it is likely that it is synergistic with the inflammatory features of ApoE^−/−^ mice and further accelerates the remodeling process. In this study, aortic lesions were associated with persistent inflammation, as demonstrated by non-invasive MPIO-αVCAM1 MR imaging. Previous studies demonstrated that VCAM-MPIO binding, evidenced as signal voids in T2* MR images, correlated well with endothelial VCAM-1 upregulation. These results were observed in different experimental settings, including the tumor–brain interface [27], systemic inflammation [15], and atherosclerosis [28]. We observed a signal void limited to the aortic valve and annulus, i.e., the irradiation target, confirming the usefulness of a targeted irradiation to the aortic valve using the MRI-based anatomic atlas. However, despite a strong association between MR findings and aortic valve irradiation, some animals presented a T2* signal void although they received no radiation, whereas some irradiated mice presented a normal MR signal. This is compatible with previous findings demonstrating early spontaneous vascular inflammation in C57BL6/J and ApoE^−/−^ mice strains. Especially, all phases of atherogenesis have been demonstrated in ApoE^−/−^ mice, from the early inflammatory response with monocyte adhesion to late fibrous caps [13]. Consequently, it is likely that the relative heterogeneity of T2* MR results reflects the temporal heterogeneity of the inflammatory process accelerated by radiation exposure.

As late development of radiation-induced aortic stenosis remains a clinical issue in patients receiving thoracic radiation therapy, this preclinical model could be useful in assessing factors that may either accelerate, like a high-fat diet, a condition associated with aortic valve remodeling, or inhibit the pathological process, like lipid-lowering therapies or inflammation modulation [29]. For example, the model might be appropriate to evaluate the involvement of NF-κB signaling, which is a mediator of various inflammatory processes involved in carcinogenesis, radiation-induced inflammation, and in the pathogenesis of various cardiovascular diseases [30]. Recently, Candellier et al. demonstrated that indoxyl-sulfate promotes osteogenic differentiation of hVIC via an activation of the AhR-NF-κB pathway [29]. Our preclinical model could be used to evaluate the impact of therapies inhibiting NF-κB signaling, including melatonin [31], aspirin [32], or metformin [33], on radiation-induced aortic remodeling.

### Limitations

The phenotypic changes of valve interstitial cells into osteoblast-like cells were not evaluated in this study. Due to the very tiny size of the aortic valve in mice, it appeared difficult to obtain a sufficient amount of valve tissue to assess the production of osteogenic factors such as osteopontin, alkaline phosphatase, or the transcription factor Runx2 using blotting techniques. However, this phenomenon was previously demonstrated by Nadlonek et al. [10] using hVICs isolated from normal aortic valves and exposed to 10 Gy irradiation.

It remains complicated to compare the dose radiation of radiation exposure in mice with the doses administrated to humans. The murine response to irradiation varies from human cellular and molecular pathways, and the complexity of radiation treatment in humans is hardly reproducible using preclinical protocols. In addition, there is no clear consensus on the radiation dose, dose rate, and fractionation that should be used in murine models of cardiac radiation toxicity [34]. The dose and fractionation chosen in this study is compatible with previous studies in the field and ensured a good tolerance of radiation exposure in mice. In clinical practice, radiation therapy is often administrated in multiple smaller radiation fractions. As the risk of radiation-induced atherosclerosis is influenced by dose fractionation [35], the effect of hypofractionation of irradiation in this animal model of aortic valve radiotoxicity remains to be investigated.

## 5. Conclusions

This study demonstrated that targeted aortic valve irradiation in ApoE^−/−^ mice resulted in the development of an aortic valve remodeling that mimics the human radiation-induced aortic valve stenosis, with a higher effect than in WT animals. This novel animal model could be useful for the preclinical assessment of therapies that may affect delayed aortic valve disease after irradiation.

## Figures and Tables

**Table 1:** Echocardiography: left ventricle dimensions at baseline and 3-month follow-up.

	Baseline		3-Month		*p*-Values			
	WT	ApoE^−/−^	WT	ApoE^−/−^	Global *p*-Value	Time Effect	Genotype Effect	Radiation Effect
IVSd (mm)	0.72 ± 0.02	0.75 ± 0.01	0.67 ± 0.02	0.68 ± 0.01	0.0009	0.0002	ns	ns
IVSs (mm)	0.89 ± 0.05	0.8 ± 0.02	0.72 ± 0.02	0.76 ± 0.03	0.01	0.0005	ns	ns
LVDd (mm)	3.70 ± 0.06	3.75 ± 0.09	3.95 ± 0.06	4.12 ± 0.09	<0.01	0.0002	ns	ns
LVDs (mm)	2.48 ± 0.07	2.59 ± 0.08	2.84 ± 0.06	3.13 ± 0.1	<0.0001	<0.0001	0.01	ns
LVPWd (mm)	0.75 ± 0.02	0.86 ± 0.04	0.82 ± 0.03	0.83 ± 0.03	ns	nd	nd	nd
LVPWs (mm)	0.98 ± 0.03	1.07 ± 0.04	0.98 ± 0.03	0.98 ± 0.04	ns	nd	nd	nd
FS (%)	33.13 ± 1.32	31.15 ± 1.08	28.27 ± 0.67	24.40 ± 1.23	<0.0001	<0.0001	<0.01	ns

Measurements were performed in 22 WT and 25 ApoE^−/−^ mice. IVS: interventricular septum, LVD: left ventricle diameter, LVPW: left ventricle posterior wall, FS: fractional shortening, d: diastole, s: systole, ns, not significant, nd: not done (i.e., post hoc tests were not performed in case of a non-significant global *p*-value for the model). Data are expressed as mean ± SEM.

**Table 2:** Functional aortic valve assessment using echocardiography at 3-month follow-up.

	WT			ApoE^−/−^			*p*-Value		
Radiation Dose	0 Gy	10 Gy	20 Gy	0 Gy	10 Gy	20 Gy	Global *p*-Value	Genotype Effect	Radiation Effect
Flow velocity (cm/s)	184 ± 5	213 ± 10	214 ± 2	261 ± 17 ^§^	308 ± 20 ^§^	312 ± 15 *^§^	<0.0001	<0.0001	<0.001
Mean gradient (mmHg)	6.33 ± 0.46	8.53 ± 0.93	8.83 ± 0.44	13.58 ± 1.69 ^§^	16.92 ± 1.71 ^§^	20.57 ± 2.04 ^§^*	<0.0001	<0.0001	<0.01
Max gradient (mmHg)	13.68 ± 0.78	18.36 ± 1.88	18.45 ± 0.4	28.05 ± 3.53 ^§^	38.57 ± 5.02 ^§^	39.75 ± 3.84 ^§^	<0.0001	<0.0001	<0.01

Peak aortic jet flow velocity, mean, and maximal trans-valvular gradients were assessed using pulse wave Doppler recordings. Data are expressed in mean ± SEM, * *p* < 0.05 vs. ApoE^−/−^ and ^§^ *p* < 0.05 vs. WT with equivalent radiation dose.

**Table 3:** Proportion of MPIO-αVCAM-1 binding using MR imaging in aortic valve leaflets and aortic sinus.

	Aortic Sinus			Aortic Valve Leaflets		
MPIO-αVCAM-1	Negative	Positive	Total	Negative	Positive	Total
RT-	4	2	6	4	2	6
RT+	4	13	17	1	16	17
Total	8	15	23	5	18	23

**Table 4:** Impact of radiation exposure on histological findings.

	WT			ApoE^−/−^			*p*-Values		
	0 Gy	10 Gy	20 Gy	0 Gy	10 Gy	20 Gy	Global	Genotype	Radiation
Leaflet area (mm^2^)	0.144 ± 0.006	0.161 ± 0.015	0.166 ± 0.015	0.115 ± 0.005	0.164 ± 0.008 *	0.195 ± 0.019 *	0.0001	ns	0.0001
Sinus tissue area (mm^2^)	0.253 ± 0.010	0.245 ± 0.009	0.217 ± 0.022	0.473 ± 0.030 ^§^	0.484 ± 0.026 ^§^	0.697 ± 0.034 *†^§^	<0.0001	<0.0001	<0.05
von Kossa area (%)	4.187 ± 11.7	3.809 ± 4.4	5.224 ± 1.62	4.224 ± 6.76	7.027 ± 10.287 *	4.027 ± 4.584 *†	<0.0001	ns	<0.0001

Mineralization was assessed using the percentage of von Kossa staining in aortic valve leaflets in 4 WT RT-, 3 WT 10 Gy, 3 WT 20 Gy, 4 ApoE^−/−^ RT-, 4 ApoE^−/−^ 10 Gy, and 3 ApoE^−/−^ 20 Gy. Data are expressed as mean ± SEM, * *p* < 0.05 vs. ApoE^−/−^ RT-, † *p* < 0.05 vs. ApoE^−/−^ 10 Gy, ^§^ *p* < 0.05 vs. dose-equivalent WT. ns, not significant.

## Data Availability

Data supporting this study will be made available to qualified researchers upon reasonable request from the corresponding author.

## Supplementary Materials

The following supporting information can be downloaded at: https://www.mdpi.com/article/10.3390/jcm12185854/s1.
