# Supplementary material for: Targeted Radiation Exposure Induces Accelerated Aortic Valve Remodeling in ApoE−/− Mice
Source: J Clin Med. 2023 Sep 8;12(18):5854. doi: 10.3390/jcm12185854 (PMC10531867; doi:10.3390/jcm12185854)
Supplement: Supplementary file 1 [file jcm-12-05854-s001.zip › jcm-2545907-supplementary.pdf]

STOP-AS Investigators

Hélène Eltchaninoff[1], Jérémy Bellien[2], Benjamin Bertrand[3], Farzin Beygui[4,5], Delphine Béziau-Gasnier[6], Ebba Brakenhielm[7], Giuseppina Caligiuri[8,9], Karine Chevreul[10,11], Frédérique Debroucker[12], Eric Durand[1], Christophe Fraschini[13], Martine Gilard[14], Bernard Iung[9], Said Kamel[15], Jamila Laschet[8], Alain Manrique[5, 16], Emmanuel Messas[17], David Messika-Zeitoun[18], Florence Pinet[19], Vincent Richard[2], Eric Saloux[4,5], Martin Thoenes[20], Christophe Tribouilloy[21], Claire Vézier[6].

1- Normandie Univ, UNIROUEN, U1096, CHU Rouen, Department of Cardiology, F-76000 Rouen, France
2- Normandie Univ, UNIROUEN, U1096, CHU Rouen, Department of Pharmacology, F-76000 Rouen, France
3- Cardiawave, Paris, France
4- CHU de Caen, Department of Cardiology, 14000 Caen, France.
5- Normandie Univ, UNICAEN, UR 4650 PSIR, GIP Cyceron, Caen 14000, France.
6- CHU Rouen, Department of Cardiology, F-76000 Rouen, France
7- Normandie Univ, UNIROUEN, U1096, F-76000 Rouen, France
8- Laboratory for Vascular Translational Science, Université de Paris, Inserm U1148, 46 rue Henri HUCHARD, Paris 75018, France.
9- Department of Cardiology, Assistance Publique-Hôpitaux de Paris, Bichat Hospital 46 rue Henri Huchard, Paris 75018 and Université de Paris, France.
10- Université de Paris, Unité UMR 1123 ECEVE, INSERM, Paris, France.
11- Health Economics Clinical Research Platform (URC Eco), AP-HP, 1 Place du Parvis Notre-Dame, 75004, Paris, France.
12- Medtronic, Boulogne-Billancourt, France.
13- Supersonic Imagine, Aix-en-Provence, France
14- Department of Cardiology, CHRU Brest, 29200 Brest, France
15- UR UPJV 7517, MP3CV, CURS, Université de Picardie Jules Verne, Avenue René Laennec, 80054, Amiens, France.
16- CHU de Caen, Department of Nuclear Medicine, 14000 Caen, France
17- Université de Paris, PARCC, INSERM, F-75015 Paris, France, vascular medicine department, AP-HP.CUP, Georges Pompidou European Hospital F-75015 Paris France.
18- Division of Cardiology, University of Ottawa, Ottawa, Ontario, Canada.
19- Inserm U1167, Institut Pasteur de Lille, Université de Lille, 59000 Lille, France.
20- Edwards Lifesciences, Nyon, Switzerland
21- Department of Cardiology, Amiens University Hospital, France; EA 7517 MP3CV Université de Picardie Jules Verne, Amiens, France.
